# Micropulse Transscleral Cyclophotocoagulation: A Hypothesis for the Ideal Parameters

**Published:** 2018

**Authors:** Facundo G. Sanchez, Juan C. Peirano-Bonomi, Tomas M. Grippo

**Affiliations:** 1 Grippo Glaucoma Center, Buenos Aires, Argentina; 2 Yale University, CT, USA

**Keywords:** Glaucoma, Micropulse Transscleral Cyclophotocoagulation, Diode Laser

## Abstract

MicroPulse transscleral cyclophotocoagulation (IRIDEX Corp., Mountain View, CA) is a novel technique that uses repetitive micropulses of active diode laser (On cycles) interspersed with resting intervals (Off cycles). It has been proposed that the OFF cycles allow thermal dissipation and, therefore, reduce collateral damage. The literature suggests that Micropulse has a better safety profile compared to traditional continuous-wave cyclophotocoagulation. However, because it is a relatively new technique, there are no clear guidelines stating the ideal laser parameters that would allow the best balance between high and sustained effectiveness with minimal side effects. This research reviewed the literature to approximate ideal parameters for single-session treatment. To simplify the comparison between studies, this study used Joules (J) as a way to standardize the energy levels employed. The reviewed clinical publications allowed reduction of these parameters to a range between 112 and 150 J of total energy, which allows a moderate IOP lowering effect of around 30% with few/no complications. An additional narrowing of the parameters was achieved after analyzing recently published experimental data. These data suggest a different mechanism of action for the Micropulse, similar to that of the pilocarpine. This effect was maximum at 150 J. Since clinical studies show few or no complications, even at those energy levels, it could be hypothesized that the ideal parameters can be located at a point closer to 150 J. This data also leads to the concept of dosimetry; the capacity to dose mTSCPC treatment based on desired IOP lowering effect and risk exposure. Further prospective studies are needed to test the proposed evidence-based hypothesis.

## INTRODUCTION

Glaucoma is one of the main causes of irreversible blindness, worldwide [1]. Historically, when hypotensive medical treatment or laser treatment of the trabecular meshwork (TM) were not enough to control the disease, traditional surgical procedures (e.g. filtering surgery, and drainage devices implantation) were contemplated. Unfortunately, they are not always effective and present a high incidence of complications of variable severity [2]. In recent years, a new group of surgical techniques, known as Minimally Invasive Glaucoma Surgeries (MIGS), have been conceived to occupy a niche in the surgical treatment of mild to moderate glaucoma [3-8]. However, although usually safe, their effectiveness and long-term performance is yet to be fully established. Regardless, when all these surgical options are exhausted, transscleral cyclophotocoagulation with diode laser becomes an option [9]. It has been proposed that this technique decreases IOP by combining two mechanisms. The first mechanism is probably the photo-destruction of the ciliary body pigmented and non-pigmented epithelium (810 nanometer diode laser wavelength is better absorbed by melanin). The second mechanism is the increase in the aqueous humor drainage through the uveoscleral pathway [10]. With this technique, severe complications are frequently reported, including hypotony, phthisis bulbi, chronic inflammation, and decreased visual acuity [11-15].

Motivated by these limitations, a modified transscleral cyclophotocoagulation technique has been developed. The infrared MicroPulse® transscleral cyclophotocoagulation (IRIDEX Corp., Mountain View, CA) is a novel technique that uses repetitive micropulses of active diode laser (On cycles), interspersed with rest intervals (Off cycles) [16]. It has been proposed that the Off periods allow for thermal dissipation, and thus, reduce collateral damage and adverse effects [13]. Energy level (i.e. total time of treatment and power), area treated, positioning of the probe, and velocity of sweeping motion are all modifiable parameters that can influence the clinical outcome of this technique. At present, given its novelty, there are no clear guidelines on how to balance these parameters to achieve high effectiveness with minimal side effects. In this article, the researchers reviewed the literature and current evidence and developed a hypothesis, in an attempt to approximate the ideal parameters for this technique.

## METHODS

A bibliographic search was performed using Pubmed, covering publications between years 2015 and 2018. A broad match combination of keywords was used, including glaucoma AND Micropulse. Twenty-eight papers were obtained. This research excluded studies that were not original articles, did not refer to glaucoma, did not employ the transscleral cyclophotocoagulation technique, were written in languages other than English, and those that did not describe the laser parameters employed. Finally, a total of nine papers remained eligible. The review was complemented using references of the cited studies, when appropriate. Finally, the researchers reviewed AGS abstracts, and ARVO abstracts published in Investigative Ophthalmology & Visual Science Journal (IOVS) during years 2017 and 2018 that were not published as papers; one of them being an experimental study (Johnstone et al, 2017).

**Micropulse versus Continuous Wave Transscleral Cyclophotocoagulation**

Micropulse is considered as equally effective yet safer than Continuous Wave Transscleral Cyclophotocoagulation (CW-TSCPC) [13]. Aquino et al. compared the efficacy and safety of Micropulse Transscleral Cyclophotocoagulation (mTSCPC) against CW-TSCPC. After 12 months of follow-up, the results showed that in the mTSCPC group, 75% of the cases (18 of 24 patients) achieved the success criteria (IOP between 6 and 21 mmHg and at least 30% decrease in baseline IOP), against 29% (7 of 24 patients) in the CW-TSCPC group (p<0.01). They reported that 46 of 48 patients completed 18 months of follow-up. No significant difference in the success rate between the two treatments was observed; the results were 52% (n = 12) for the Micropulse and 30% (n = 7) for the CW-TSCPC method (P = 0.13). The mean baseline IOP, at 18 months of follow-up, decreased by 45% in both groups (P = 0.70), from a baseline of 36.5mmHg (29.5 to 16.5) in the mTSCPC group and 35.0mmHg (29.5 to 46.5) in the CW-TSCPC (P = 0.50). There were no significant differences in the IOP reduction between the two groups (P = 0.70). There were no significant differences in the need for retreatment (P = 0.36). There was also no difference in the decrease of IOP lowering medications (P = 0.88), this being two to one drug in both groups (P < 0.01). However, the complication rate was higher with the CW-TSCPC method (P = 0.01). Prolonged hypotonia (IOP ≤ 5mmHg for more than six months) was observed in five eyes treated with CW-TSCPC versus none in the MicroPulse group, visual acuity decrease was found in 9% (2/23) of the CW-TSCPC group versus 4% (1/23) in case of the mTSCPC, prolonged inflammation of the anterior chamber was observed in 30% (7/23) versus 4% (1/23), phthisis bulbi 4% (1/23) versus 0%, and scleral thinning 17% (4/23) versus 4% (1/23), respectively [13]. Similar results were observed in a comparative study between CW-TSCPC and mTSCPC, on 45 eyes of 36 pediatric patients. The success rate was higher in the mTSCPC group (71% versus 46% in the CW-TSCPC group) yet the difference was not significant (P = 0.1). No significant complications were noted in the mTSCPC group, whereas in the CW-TSCPC group, one eye developed phthisis bulbi and two eyes had severe pain and uveitis (P = 0.3) [17].

**Mechanism of Action**

The mechanism of action of this technique is not yet fully elucidated. Traditional diode laser has a greater absorption in melanin-containing tissues [9, 18]. Although the adjacent non-pigmented tissues absorb less direct energy from the laser, they still exhibit collateral damage [18]. This occurs because it is impossible to avoid an indirect transfer of caloric energy from the targeted pigmented epithelium and dissipate it before reaching coagulation temperatures [19]. Clinical and experimental studies propose that the OFF periods in the Micropulse restrict the accumulation of caloric energy in the tissues adjacent to the pigmented epithelium. This allows thermal dissipation, preventing from reaching coagulation temperatures and, therefore, reducing collateral damage [16, 20, 21]. A second component for the mechanism of action, involving an increase in the aqueous humor drainage through the uveoscleral pathway, has also been reported [10]. A third mechanism of action for the Micropulse has recently been proposed by Johnstone [22] et al., after an experimental study on monkeys (m. Fascicularis) [22]. According to the authors, the pigmented epithelium is not necessarily involved in the mechanism of action. In contrast, Micropulse would actually act on the longitudinal fibers of the Ciliary muscle (CM), causing a displacement of the Scleral Spur (SS) in a posterior and inward direction, which in turn modifies the configuration of the TM and the outflow tract of the aqueous humor. This effect is similar to that of pilocarpine, which causes enlargement of the trabecular spaces and expansion of the Schlemm’s canal area, reducing the tendency towards collapse or narrowing of the canal lumen, thus, facilitating the drainage of aqueous humor [23]. It is difficult to conclude at this point if one of these suggested mechanisms of action has the most effect on IOP or whether the IOP lowering effect of this technique is in fact a combination of all mechanisms.

**Surgical Technique**

Laser treatment is applied over the 360 degrees of the eye, sparing -or not- the three and nine o’clock position. Some physicians opt for a fast-sweeping motion of about 10 seconds back-and-forth over 180 degrees, others use a slow-sweeping motion of about one-minute over the same distance, or somewhat in between [24].

The probe has a notch that is oriented towards the central cornea and its base rests close to the conjunctival limbus or a few millimeters posterior. The current probe was designed to allow for precise positioning of the fiber-optic tip at 3 mm posterior to the limbus [13, 16].

**Ideal Parameters: where are we Standing?**

Despite promising initial results, the ideal parameters for the Micropulse, to obtain the best balance of efficacy/safety, have not yet been established. Nevertheless, at this point, the miscellaneous data currently available on the literature can help approximate these parameters. Due to considerable variability in laser settings within published studies, it is useful to convert the total energy used in each of the settings to Joules in order to facilitate interstudy comparison, as introduced by Murray Johnstone et al. in their experimental study [22]. Joules (J) = power in Watts (W) x total treatment duration in seconds (s) x ON cycle (31.3%). This would exclude other potential variables from the equation, such as velocity of sweeping motion and the positioning distance of the probe from the limbus.

**Figure 1:**
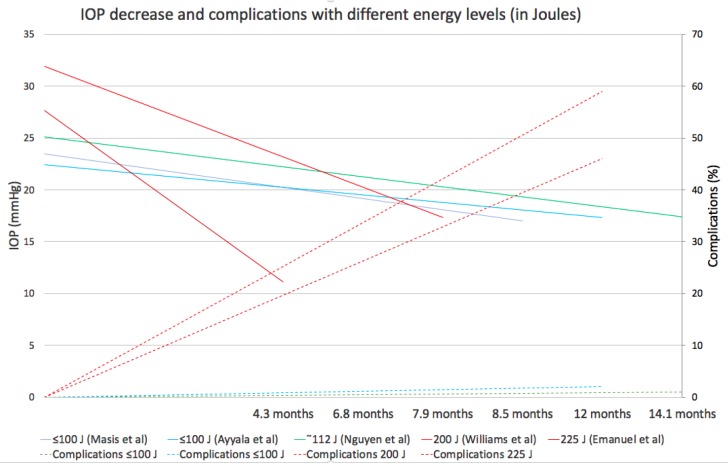
Clinical Outcomes with Variable Energy Levels. X-axis: Follow-up Time. Continuous Lines on Y-axis: IOP Decrease. Secondary Axis (Dashed Lines): Complications (%). Warm, Green and Cold Colors Represent High, Medium and Low Energy Levels, Respectively. Abbreviations: J: Joules.

**Figure 2:**
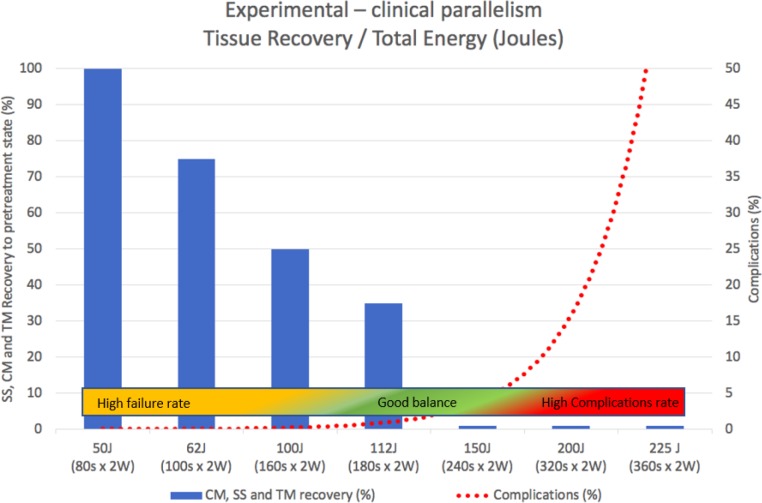
Experimental and Clinical Data Overlap. X-axis shows Increasing Energy Levels in Joules. Blue Bars on the Y-axis Summarizes the Progressively Less Recoil/Relaxation of SS, CM, and TM observed in the Experimental Study (Johnstone et al). Secondary axis represents Clinical Complications with Increasing Energy Levels Reported in the Literature. The Colored Bar Illustrates Evidence-based Hypothesis of the Best Balance Efficacy/Safety. Abbreviations: J: Joules; W: Watts; CM: Ciliary Muscle; SS: Scleral Spur; TM: Trabecular Meshwork.

Fig 1 illustrates five representative studies that used different energy levels, mainly by varying treatment duration. Continuous, warm-colored lines represent higher energy levels (200 to 225 J), while green is for mid-range levels (112 to 140 J) and cold colors are for low-energy levels (≤100J). It is possible to infer a positive relationship between total energy and IOP decrease. Williams et al. [25] and Emanuel et al. [24] used up to 200 J and 225 J of energy (320 and 360s x 2W x 31.3% ON cycle) and obtained an IOP decrease from baseline of 46% and 60%, respectively. However, complications soared to more than 45% in both studies (dashed lines represent complications). Persistent hypotonia, postoperative inflammation >3 months, and loss of 2+ Snellen lines were the most common. Indeed, the upper level of total energy that can be applied is mainly limited by the emergence of complications. There are a few reports that applied mid-range energy levels of around 112 to 140 J (180s x 2 to 2.5W and 31.3% ON cycle). They obtained a moderate IOP decrease of approximately 30%, without complications [26-28] for at least 12 months. Other studies that used relatively low energy levels, ≤100 J (≤ 160s x 2W x 31.3 ON cycle), were moderately effective (around 30% IOP decrease) in the short-term (~1 month) without complications, yet in many cases, more than one laser session (up to three) was required to maintain the effect in the mid-term [18, 28, 29], leading some authors to abandon them for insufficient results [25]. The study by Sanchez FG et al. was in concordance with the literature [30]. The researchers applied energy levels within the 62 to 112 J range (varying only the treatment duration, power fixed at 2W, and ON cycle at 31.3%) on 22 eyes of 17 patients (mostly congenital and pseudoexfoliation types), with over six months of follow-up with only one treatment session per eye. Overall success was low (27.3%). However, patients treated with higher energy levels (112 J) obtained up to 75% success and an IOP decrease from baseline of 34%. All patients that received the lowest energy level (62 J) failed. No complications were observed in any of the cases. Taken together, all these clinical data indicate a good balance of efficacy/safety, ranging from 112 to 150 J, allowing a moderate IOP decrease of about 30% with few/no complications. From this point on, the data of clinical origin do not allow further approximation of the ideal parameters for the mTSCPC. Fortunately, the very interesting experimental work in enucleated m. Fascicularis eyes presented by Johnstone et al. [22] provides key data with untapped potential that may help better understand the clinical evidence so far. As it was previously described under ‘mechanism of action’, the authors propose a pilocarpine-like effect for the Micropulse as its main mechanism of action, rather than photocoagulation of the pigmented ciliary epithelium. Therefore, they designed the experiment to evaluate recoil/relaxation in the pre-treatment state of the CM, SS, and TM, after different levels of total energy was delivered with the mTSCPC. Fig 2 shows the recovery percentage (y-axis) observed in their experiment with increasing total energy in Joules (x-axis). An equivalent of Joules expressed in *seconds* x *power* was added to facilitate the interpretation.

An almost complete recovery/relaxation (i.e. complete loss of the pilocarpine-like effect) was observed at lower energy levels of less than 62 J (equivalent to a laser setting of 100s x 2W x 31.3% ON cycle). Progressively less recovery was observed as more energy was applied. Above a considerably higher energy level (150 J), no recovery was observed (i.e. full permanence of the effect). This is equivalent to a laser setting of 240s x 2W x 31.3% ON cycle. Superimposed is a red dotted line representing the emergence of adverse effects reported so far in clinical studies. A colored bar was added, summarizing the clinical results with different energy levels in the literature. The experimental data suggests that the pilocarpine-like effect explains at least part of the clinical response observed with different energy levels. Based on this, to fully benefit from this mechanism, the energy level should be increased to nearly 150 J (equivalent to 240s x 2W x 31,3%) [22].

Beyond 150 J, significant side effects start to appear, while no additional pilocarpine-like effect is added. Taken together, the clinical and experimental results suggest a sweet spot between 112 to 150 J. When the clinical and the experimental results are combined, it is possible to redefine the sweet spot more precisely at energy levels closer to 150 J to maximize the pilocarpine-like effect. In this way, with energy levels closer to 150 J, maximum benefit could be obtained from the combination of the multiple mechanisms of action proposed for the Micropulse, without triggering the complication rate. Although above 200 J the percentage of IOP reduction is greater (approximately 40% to 60% from baseline), moderate to severe complications increase considerably [24, 25]. A possible explanation may be that the extra IOP lowering effect mainly depends, at this level, on cyclodestruction.

**Re-treatment**

When it comes to using this new therapeutic technology, two surgical approaches are possible. One treatment is described above with the expectation of achieving success or the possibility of repeated treatments with lower energy levels to achieve target pressure. There is little published evidence on re-treatments. Aquino et al. [13] and Tan et al. [16] used shorter treatment durations of 100s x 2W (62.5J), yet performed a second or a third session when the first session had failed. From a total of 23 patients treated with the Micropulse, Aquino et al. needed to re-treat 11 patients (48%), whose IOP remained uncontrolled after a mean of 6.8 months (range two to 17 months). They performed a second session on seven patients and a third session on four. Even after the third session, those four eyes (all neovascular glaucomas) failed again. 

Comparably, Tan et al. needed to re-treat 14 out of 40 eyes (35%). From those 14 eyes, nine failed again after the second treatment yet did not undergo a third session. No significant complications were observed in any of the studies.

**Increased Risk in Certain Populations**

Radhakrishnan et al. reported higher odds of persistent mydriasis in the Asian race (OR 13.07, P < 0.001) and phakic status (OR 3.12, P = 0.014) in a 144-patient cohort [31]. Williams et al. stated that it is reasonable to use shorter treatment times in heavily pigmented patients with the understanding that repeat treatment may be needed later, should this initial approach be ineffective, and considering the significantly higher odds of prolonged inflammation found in this population (OR 3.61, 95% CI 1.27-10.23; P = 0.02). In patients with good vision at baseline (given the non-infrequent incidence of vision loss in their study), 13 patients showed loss of 2+ lines of best-corrected visual acuity for ≥ 3 months (16.5%) [25]. Individualizing treatments based on patients and disease characteristics is the next step in the ongoing development of this technology. 

## CONCLUSION

The available data, although scarce and difficult to compare because of varying definitions of success criteria and use of laser parameters, suggest that a good balance of efficacy/safety can be narrowed to an intermediate amount of total energy of approximately 112 to 150 J. This would allow an adequate IOP decrease with few/no complications, opening the possibility and initial rationale to dose the treatment based on desired IOP lowering effect and risk exposure. Prospective comparative studies, with homogeneous success criteria definitions and longer follow-up periods, are necessary to precisely determine Micropulse’s ideal parameters, especially evaluating the individual characteristics of each patient and their glaucoma. It would be interesting to also evaluate the impact of re-treatment, probe motion velocity, and probe positioning on clinical outcomes.
